# A single-institution study of concordance of pathological diagnoses for interstitial lung diseases between pre-transplantation surgical lung biopsies and lung explants

**DOI:** 10.1186/s12890-019-0778-x

**Published:** 2019-01-21

**Authors:** Tanmay S. Panchabhai, Andrea Valeria Arrossi, Kristin B. Highland, Debabrata Bandyopadhyay, Daniel A. Culver, Marie M. Budev, Carol F. Farver

**Affiliations:** 1 Norton Thoracic Institute, St. Joseph’s Hospital and Medical Center, Phoenix, AZ USA; 2 Department of Pathology, Pathology and Laboratory Medicine Institute, Cleveland Clinic, Cleveland, OH USA; 3 Department of Pulmonary Medicine, Respiratory Institute, Cleveland Clinic, Cleveland, OH USA; 4 Department of Thoracic Medicine, Geisinger Medical Center, Danville, PA USA

**Keywords:** Surgical lung biopsy, Explant pathology, Interstitial lung disease, Lung transplantation, Discordant diagnosis

## Abstract

**Background:**

By comparing diagnoses made by pre-transplant surgical lung biopsy (SLB) and the final pathologic diagnosis of the explanted pathology (EP), we aimed to study the factors that could impact pathologic diagnoses in patients with interstitial lung disease (ILD).

**Methods:**

We retrospectively reviewed the lung transplant database at Cleveland Clinic [01/01/2006–12/31/2013] to include all lung transplant recipients with a prior diagnosis of ILD. Two pulmonary pathologists independently reviewed each SLB and lung explant. The diagnoses were labeled as concordant (same diagnosis on SLB and explant) or discordant (diagnosis on SLB and explant were different) by consensus.

**Results:**

Of 389 patients transplanted for ILD, 217 had an SLB before transplant. Pathological diagnoses were concordant in 190 patients (87.6%) [165 UIP (86.8%), 13 NSIP (6.8%), 8 CHP (4.2%) and 4 other diagnoses (2.1%). In 27 cases (12.4%), the diagnosis on SLB differed from EP. 8/27 were diagnosed with UIP on SLB and of these, 5 were re-classified as NSIP. 14/19 (73.7%) patients with a SLB diagnosis “other than UIP” were re-categorized as UIP based on explant. Discordant cases had a greater time between SLB and EP than concordant cases (1553 days vs 1248 days).

**Conclusions:**

The pathologic diagnosis of ILD by SLB prior to lung transplant is accurate in most patients, but may be misleading in a small subset of patients. The majority of discordant cases that were reclassified as UIP could be due to a sampling error, or perhaps, an increased time from the date of the SLB to transplant. Future studies examining how multidisciplinary consensus diagnosis affects this discordance are necessary.

## Background

The management of interstitial lung disease (ILD) has undergone a significant paradigm shift over the past decade and with this shift has come an increased demand for accurate diagnosis of ILD. This is especially important for idiopathic pulmonary fibrosis (IPF), which has the worst prognosis and few potential treatment options to slow the progression of disease [1]. Moreover, a definitive diagnosis of IPF also prompts consideration and referral for lung transplantation [1]. The 2015 ATS/ERS/JRS/ALAT Guidelines for diagnosis and management of IPF suggest high-resolution computed tomography (HRCT) for diagnosis [1]. The surgical lung biopsy (SLB), once the gold standard for ILD diagnosis, has given way to “probable” and “definitive” patterns shown to be consistent with a pathologic diagnosis of usual interstitial pneumonia (UIP) on HRCT. A multidisciplinary consensus diagnosis (whether based on SLB pathology or not) is now considered the gold standard for the diagnosis of ILD [2]. The utility of the multidisciplinary consensus diagnosis has been recommended to aid the following scenarios: 1) When clinical context or CT pattern or both are indeterminate, to aid in decision making for additional work-up including bronchoscopy, SLB, etc.; 2) After SLB to evaluate clinical, radiological and pathological findings; 3) Re-review patients in whom the clinical course is discordant with a prior multidisciplinary diagnosis; or 4) When diagnostic tissue is not available and to consider a working diagnosis [2]. SLB was considered to be the gold standard prior to current treatment guidelines [1, 2] and still forms the basis of obtaining diagnostic tissue to aid multidisciplinary consensus [2]. However, conflicting results have been reported regarding the efficacy of single or multiple SLBs on the diagnostic yield for ILD [3, 4].

The pathological diagnoses of UIP and non-specific interstitial pneumonia (NSIP) have received the most attention in prior studies due to their effects on prognosis and survival [5]. Previous research has shown that different patterns may be seen in separate samples obtained from the same patient, resulting in the proposed nomenclature of concordant UIP, discordant UIP, and concordant NSIP [6]. Similarly, early studies comparing diagnoses between SLB and lung explants have demonstrated the presence of NSIP-like pattern in the lungs of patients ultimately diagnosed with UIP [7, 8]. Factors such as the number and areas of the samples, time from surgical lung biopsy to transplantation, and interobserver variability among the reviewing pathologists have been studied in prior reports [6, 9]. Only one study to date has compared the diagnoses on pre-transplantation SLB and explanted lung [7], while 3 others have compared pre-transplant clinical diagnoses to pathological diagnoses made after reviewing the lung explant [10–12]. All 4 studies have found discordance rates of 10–20% [7, 10–12]. These studies were carried out before the Lung Allocation Score (LAS) system was introduced in 2005, after which the number of lung transplant recipients for ILD (most commonly IPF) has significantly increased, changing the composition of the patient population reviewed [13]. These studies were done prior to the integration of the current IPF guidelines into clinical practice, which emphasize the role of HRCT in the diagnostic algorithm [1, 7, 10–12]. Finally, the increased use of pre-transplant mechanical ventilation or extracorporeal membrane oxygenation (ECMO), which may lead to significant acute superimposed pathologic findings in the explanted lung, can make the diagnosis of the underlying ILD on explant even more challenging.

The purpose of this study was to retrospectively evaluate the concordance rate between the pathologic diagnoses made on SLBs and the explant pathology (EP) specimens of lung transplant recipients and to review the possible factors that affected discordant diagnoses.

## Methods

### Data collection

After approval by the Cleveland Clinic Institutional Review Board (IRB # 14–1489), we retrospectively reviewed the lung transplant database at Cleveland Clinic, Cleveland, OH for all patients who underwent lung and heart–lung transplantations from 01/01/2006–12/31/2013. All patients listed for lung transplantation for a clinical diagnosis of ILD were included. Medical record numbers were retrieved to extract the following data from electronic medical records: patient demographics (e.g., age, sex, and body mass index), transplant date, transplant type, pre-transplant SLB diagnosis, site, number of lobes sampled, time from SLB to lung transplantation and the pathologic diagnostic review (microscopic slides read by Cleveland Clinic pathologists versus outside report review of outside pathologists’ slide review).

### Pathological review

Two pathologists (AVA and CFF), both with specialty training in pulmonary pathology, independently reviewed the pathology from all explanted lungs of patients transplanted for ILD and for all pre-transplant SLB where microscopic slides were available, including SLBs performed at outside hospitals. When the original slides of outside SLBs were unavailable for histopathological review, the pathologic diagnosis rendered by the outside pathologist was accepted. Histopathological review between SLB and EP occurred at different time intervals to avoid any confounding bias. ILD was diagnosed according to previously defined diagnostic criteria for usual interstitial pneumonia (UIP), non-specific interstitial pneumonia, cellular or fibrosing type (NSIP-cellular or fibrosing type), desquamative interstitial pneumonia (DIP), chronic hypersensitivity pneumonitis (CHP), acute lung injury or organizing acute lung injury (ALI/org ALI) [14], cryptogenic organizing pneumonia/organizing pneumonia (COP/OP), constrictive bronchiolitis (CB), connective tissue disease-associated interstitial lung disease (CTD-ILD), and interstitial lung disease–not otherwise specified (ILD-NOS) [14].

Discordant UIP (separate UIP and NSIP patterns in different samples of the same patient) has been reported to have outcomes similar to concordant UIP (same UIP pattern in different samples) and significantly worse when compared with concordant NSIP in SLBs [6, 9]. Hence, we conferred a diagnosis of UIP in SLBs if UIP pattern was seen in any of the sampled lobes. In 13 of the 217 cases (5.9%), the pathologists disagreed on the initial review of either the SLB or the explant pathology (EP). In these instances, the case was re-reviewed in order to obtain a mutual consensus. Subjects were categorized as having concordant diagnoses if the diagnosis of SLB and EP were similar. They were categorized as having discordant diagnoses if the final diagnoses of the SLBs and the EP differed. Likewise, the discordant diagnoses category was conferred if there was not a definitive diagnosis (i.e. ILD-NOS or end-stage fibrosis) given to the SLB and a definitive diagnosis was rendered on the EP, or vice versa. The concordant diagnosis category was accepted if the SLB was definitive, but a definitive diagnosis could not be reached on the EP due to the presence of prominent acute lung injury that obscured the underlying fibrotic lung disease.

## Results

### Demographics

Between 01/01/2006 and 12/31/2013, 774 lung transplantations were performed at Cleveland Clinic. Indications for lung transplantation were: ILD (*n* = 389, 50.3%), chronic obstructive pulmonary disease (*n* = 193, 24.9%), cystic fibrosis (*n* = 84, 10.9%), pulmonary arterial hypertension (*n* = 41, 5.3%) and others (*n* = 67, 8.7%). Of the 389 patients transplanted for ILD, 217 (55.8%) underwent SLB before lung transplantation and were the patients included in our study. There were 164 men and 53 women and the mean age at the time of transplantation was 58.90 years. Forty-four patients received right single lung transplant, 65 received left single lung transplant, 107 received bilateral sequential lung transplant, and 1 patient received a heart–lung transplant.

Of the 217 SLBs reviewed, 131 SLBs were from the right side and 86 SLBs were from the left. SLBs were obtained from one lobe in 26 patients (11.9%), from 2 lobes in 127 patients (58.5%) and from 3 lobes in 24 patients (11.0%). Information regarding lobe sampling was not obtained for 40 patients (18.4%). The overall mean time interval between the SLB and transplantation was 1293 days (range 15–8124 days). The distribution of laterality of SLB among the lung transplant recipients was as follows: of the 108 BSLT, 64 had right SLB and 44 had left SLB; of the 44 RSLT, 25 had right SLB and 19 had left SLB and of the 65 LSLT, 42 had right SLB and 23 had left SLB.

### Pathologic diagnoses (Tables 1 and 2; Figs. 1 and 2)

**Fig. 1:**
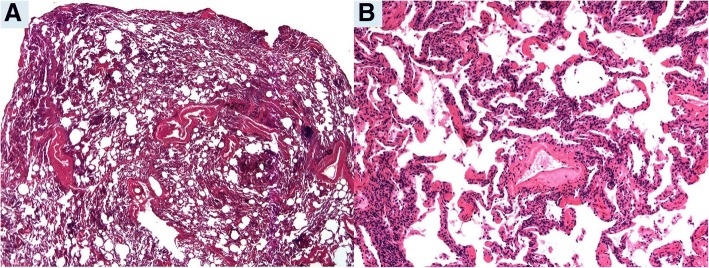
Pre-transplantation surgical lung biopsy (SLB) in Patient 7: **a**. SLB revealing chronic inflammation without evidence of fibrosis diffusely involving the lung consistent with non-specific interstitial pneumonia, cellular type. (Hematoxylin and eosin, 12.5x). **b**. Lymphocytic infiltrate expands the alveolar walls (Hematoxylin and eosin, 100x)

**Fig. 2:**
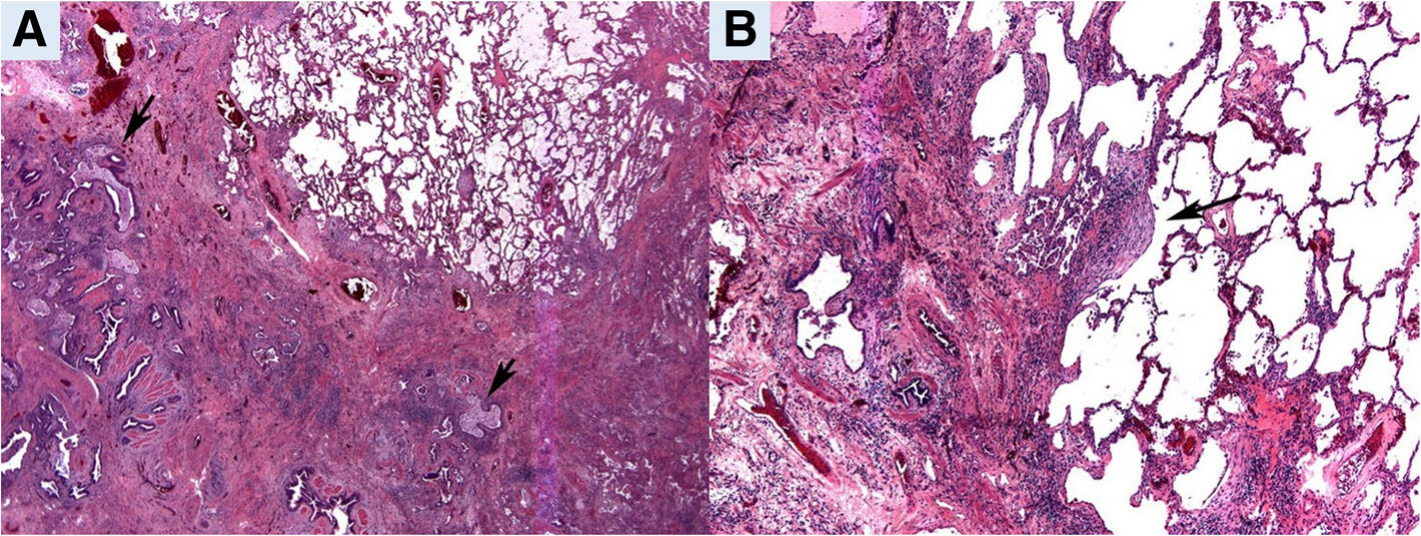
Explant pneumonectomy in Patient 7: **a**. Patchy interstitial fibrosis with honeycomb changes (arrow) consistent with usual interstitial pneumonia (UIP). (Hematoxylin and eosin, 12.5x). **b**. Fibroblastic focus (arrow) confirms UIP diagnosis. (Hematoxylin and eosin, 100x)

The SLB diagnoses included 179 UIP (82.4%), 18 NSIP (8.2%), 10 CHP (4.6%) and 10 miscellaneous (4.6%). Concordance between SLB and EP diagnoses occurred in 190 (87.6%) cases and included 165 UIP (86.8%; 3 cases classified as end-stage interstitial fibrosis with possible UIP pattern), 13 NSIP (6.8%; 3 cases classified as end-stage interstitial fibrosis with possible NSIP pattern), 8 CHP (4.2%) and 4 miscellaneous diagnoses. In 27 cases (12.4%), the diagnoses between SLB and EP were discordant. The final diagnoses based on EP review of the 27 discordant cases were: 14 UIP, 6 NSIP, 2 CHP, 3 ILD-NOS, 1 CTD-ILD, and 1 end-stage fibrosis with pulmonary alveolar proteinosis-like features, suggestive of an environmental etiology. Among the discordant cases, the most common discordances included NSIP on SLB with final diagnosis of UIP on EP (*n* = 7) and UIP on SLB with final diagnosis of NSIP on EP (*n* = 5) (Figs. 1 and 2). Together, these two types of discordances comprised 44% of the discordant cases.

**Table 1:** Discordant diagnoses between surgical lung biopsies and lung explants (*n* = 27)

No.	Diagnosis on SLB	SLB location	SLB No. of lobes	Diagnosis on Explant	Explanted lung
1	Fibrotic NSIP	Right	2	UIP	Left
2	UIP	Left	N/A	ILD-NOS	Both
3	UIP	Left	N/A	CHP	Left
4	CHP	Right	N/A	UIP	Both
5	NSIP	Left	2	UIP	Both
6	COP	Right	N/A	UIP	Both
7	NSIP	Left	2	UIP	Both
8	DAD	Right	1	NSIP	Both
9	NSIP	Right	2	UIP	Both
10	DIP	Left	N/A	UIP	Both
11	NSIP	Left	2	ILD-NOS	Both
12	UIP	Right	2	Fibrotic NSIP	Both
13	ALI	Right	2	UIP	Both
14	Chronic Bronchiolitis	Right	3	UIP	Both
15	UIP	Right	2	Fibrotic NSIP	Right
16	NSIP	Right	2	UIP	Both
17	UIP	Left	N/A	NSIP	Both
18	BOOP	Right	2	ILD-NOS	Both
19	UIP	Right	N/A	Fibrotic NSIP	Left
20	BOOP	Left	2	UIP	Left
21	Chronic Bronchiolitis	Right	1	CTD-ILD with PAH	Both
22	NSIP	Left	2	UIP	Right
23	NSIP	Right	2	UIP	Both
24	UIP	Right	2	NSIP	Right
25	UIP	Left	2	End-stage IF, likely environmental	Both
26	NSIP	Right	2	CHP	Left
27	ILD-NOS	Left	N/A	UIP	Both

Abbreviations: *ALI* acute lung injury, *BOOP* bronchiolitis obliterans organizing pneumonia, *CHP* chronic hypersensitivity pneumonitis, *COP* cryptogenic organizing pneumonia, *CTD-ILD* connective tissue disease-associated interstitial lung disease, *DAD* diffuse alveolar damage, *DIP* desquamative interstitial pneumonia, *IF* idiopathic fibrosis, *ILD-NOS* interstitial lung disease–not otherwise specified, *N/A* not available, *NSIP* non-specific interstitial pneumonia, *PAH* pulmonary arterial hypertension, *SLB* surgical lung biopsy, *UIP* usual interstitial pneumonia

**Table 2:** Discordant diagnoses between surgical lung biopsies and lung explants (*n* = 27)

	Definitive pathological diagnoses from lung explants				
Pathological diagnoses made on surgical lung biopsies	UIP	NSIP	CHP	ILD-NOS^a^	Other diagnoses
UIP		5	1	1	1^b^
NSIP	7		1	1	
CHP	1				
ILD-NOS^a^	1				
ALI	1				
COP/BOOP	2			1	
DIP	1				
DAD		1			
Chronic bronchiolitis	1				1^c^
Total *n* = 27	14	6	2	3	2

^a^Interstitial fibrosis which is not pathologically classifiable

^b^ Endstage interstitial fibrosis, likely environmental etiology

^c^ Connective tissue disease associated ILD with pulmonary hypertension

Abbreviations: *ALI* acute lung injury, *BOOP* bronchiolitis obliterans organizing pneumonia, *CHP* chronic hypersensitivity pneumonitis, *COP* cryptogenic organizing pneumonia, *DAD* diffuse alveolar damage, *DIP* desquamative interstitial pneumonia, *ILD-NOS* interstitial lung disease–not otherwise specified, *NSIP* non-specific interstitial pneumonia, *UIP* usual interstitial pneumonia

We focused on the following variables in the present dataset: number of lobes evaluated, the pathologic review by Cleveland Clinic pulmonary pathologists versus outside pathologists, and the time interval between the SLB and the lung transplantation (Table 3). In the 190 concordant cases, 24 cases had 1 lobe sampled, 111 had 2 lobes sampled, 23 had 3 lobes sampled. In 32 cases, the number of lobes sampled was unknown. The mean time from SLB to explant in these 190 cases was 1248 days (range 15–6855 days). Of the 27 patients for whom diagnoses were discordant between SLB and EP, 2 had 1 lobe sampled, 16 had 2 lobes sampled, 1 had 3 lobes sampled, and the number of samples was unknown in 8 patients. The mean duration of SLB to transplant in these discordant patients was 1553 days (range 29–8124 days). There were 64 SLB diagnoses by Cleveland Clinic pathologists with 5 discordances (7.8%) and 153 diagnoses by outside pathologists with 19 discordances (12.4%).

**Table 3:** Concordance versus Discordance by Number of Lobes Sampled, Type of Pathology Review and Time from SLB to Transplantation

	Concordant	Discordant	Total
Number of Lobes Sampled			
1 Lobe	24	2	26
2 Lobes	111	16	127
3 Lobes	23	1	24
Unknown	32	8	40
	190	27	217
Outside Review (OSR) versus Cleveland Clinic (CC) Pathology Review			
OSR	134	19	153
CC	59	5	64
Total	190	27	217
Time from SLB to Transplantation (Days)			
	190	27	217
Minimum-Maximum Days to transplant	2–6855	263–8124	
Average Days	1256.2	1560.3	

## Discussion

SLB has long been recognized as the gold standard when making a diagnosis of ILD. With advances in management and strict criteria for treatment options, more patients with suspected ILD are undergoing SLB for definitive diagnosis, especially those with atypical clinical and/or radiologic findings. Importantly, a diagnosis of UIP has significant impact on the prognosis and management of patients given the availability of anti-fibrotic therapies such as nintedanib [15] and pirfenidone [16]. Moreover, per guidelines from the International Society of Heart and Lung Transplantation, patients with a diagnosis of UIP should be referred to a lung transplantation center for evaluation of disease progression and candidacy for lung transplantation [17]. However, conflicting data exist regarding the ultimate accuracy of SLB and the factors that may affect it, including the site of the biopsy (i.e., if a more fibrotic area is sampled and if less involved lung is sampled) and number of samples taken [3, 4]. Studies comparing pre-transplant SLB diagnosis with EP pathology provide insight into the possible sources of error for prior clinical-pathological diagnoses.

Samples with an NSIP pattern may be present in patients ultimately diagnosed with UIP [7, 8]. Flaherty et al. defined discordance in the diagnosis of UIP and NSIP from video-assisted thoracoscopic surgery (VATS) samples from the same patient, standardizing terminology with descriptors that include concordant-UIP, discordant-UIP and concordant-NSIP [6]. Likewise, discordance has also been reported in 10–20% of prior studies that compare pre-transplant diagnosis via SLB and diagnosis based on EP [10–12].

This study represents the largest single-institution study of factors that may affect the discordance rate between SLB and EP pathologic diagnosis. Similar to earlier studies, our results show discordant pathologic diagnosis between SLB and EP in 12.4% of patients. The most common discordant diagnosis included NSIP found in SLB with UIP found in the EP (*n* = 5) and UIP found in the SLB with NSIP in the EP (*n* = 7). Our data support our hypothesis that site of SLB, number of lobes sampled and time between SLB and EP could influence concordance. Furthermore, the fewest number of discordant cases were found when 3 lobes were sampled. In those patients who underwent single lung transplant (RSLT = 44, LSLT = 65), 61 had SLBs from the opposite lung (19 left SLB of 44 RSLT and 42 right SLB of 65 LSLT). Of these, only 4 had discordant diagnoses. Our data further expand these findings to include other types of ILDS. Interestingly, 14/19 (73.7%) subjects with a SLB diagnosis “other than UIP” ended up being re-categorized as UIP based on explant. Patients with discordant SLB and EP diagnosis had a longer duration from SLB to EP (1553 vs 1248 days). This suggests a “sampling” error that could be attributed to the size of the biopsies and the place or number of lobes sampled (since discordances were least when 3 lobes were sampled).

Interobserver variability among pathologists may account for discordant diagnoses on SLB with EP. In our study, of the 64 biopsies reviewed by Cleveland Clinic pathologists, there were 5 discordant diagnoses (7.8%). Of the 153 biopsies that were read by outside pathologists, there were 19 discordant diagnoses (12.4%). The difference in these rates of discordance may be explained by 1) both Cleveland Clinic pathologists had previous fellowship training in pulmonary pathology and have worked at an institution with a high volume of lung pathology, and 2) the Cleveland Clinic pulmonary pathologists had clinical and radiologic information during their pathologic evaluation of the SLBs. Nonetheless, the difference in discordance is small. This could perhaps be attributed to the fact that many of the reports of the outside SLBs were reviewed by known pulmonary pathology-trained pathologists with national consultation services. Also, in medical centers where VATS biopsies are performed for ILD, it may indicate a high level of pulmonary pathology skills developed by general surgical pathologists, who have the opportunity to evaluate these biopsies.

## Conclusions

New medical therapies are emerging for treatment of patients with ILD. Therefore, it is important that accurate diagnoses are made to ensure selection of patients who will benefit most from these therapies and to identify those who should be immediately referred for lung transplantation. Our study suggests that increasing the number of SLBs taken will increase the odds of a correct/concordant diagnosis. Nevertheless, clinicians should be aware that UIP and NSIP are susceptible to inaccurate pathologic diagnoses. Moreover, with newer techniques such as bronchoscopic cryobiopsies being evaluated for the diagnosis of ILD, the issue of concordance and discordance with explant pathology (arguably the gold standard) becomes even more critical as the size of cryobiopsies is smaller than SLB. In addition, cryobiopsies are not routinely being performed from multiple lobes, based on recommendations for SLB in the ILD diagnostic algorithms. Future studies hence should ideally include comparisons between cryobiopsies, SLB, and multidisciplinary consensus diagnosis as compared to explant diagnosis. Such studies evaluating the effect of a multidisciplinary consensus diagnosis with recommendations of optimal surgical site sampling may reduce discordance between pre-transplantation SLB and explant pathologic diagnosis, improving the management and outcomes in these patients.

## Abbreviations

ALI: Acute lung injury
CHP: Chronic hypersensitivity pneumonitis
CTD-ILD: Connective tissue disease-associated ILD
DLT: Double lung transplant
ECMO: Extracorporeal membrane oxygenation
EP: Explant pathology
HRCT: High resolution computed tomography
ILD: Interstitial lung disease
ILD-NOS: Interstitial lung disease – not otherwise specified
IPF: Idiopathic pulmonary fibrosis
LAS: Lung Allocation Score
LSLT: Left single lung transplant
NSIP: Nonspecific interstitial pneumonia
RSLT: Right single lung transplant
SLB: Surgical lung biopsy
UIP: Usual interstitial pneumonia
VATS: Video-assisted thoracoscopic surgery
